# Bias in sex ratios and polyandry rate in reproduction of *Leptinotarsa decemlineata*

**DOI:** 10.1038/s41598-022-26177-z

**Published:** 2022-12-14

**Authors:** Vladimíra Sedláková, Jitka Stará, Daniela Čílová, Martina Melounová, Jakub Vašek, Pavel Vejl, Petr Doležal, František Kocourek, Ervín Hausvater, Petr Sedlák

**Affiliations:** 1 Department of Genetics and Breeding, Faculty of Agrobiology, Food and Natural Resources, Czech University of Life Sciences Prague, Kamýcká 129, 16500 Prague 6 Suchdol, Czech Republic; 2 Team of Integrated Crop Protection Against Insect Pests, Crop Research Institute, Drnovská 507/73, 16106 Prague 6 Ruzyně, Czech Republic; 3 Department of Potato Protection, Potato Research Institute Havlíčkův Brod, Ltd., Dobrovského 2366, 58001 Havlíčkův Brod, Czech Republic

**Keywords:** Population genetics, Entomology

## Abstract

The Colorado potato beetle (CPB, *Leptinotarsa decemlineata* Slechtd.) is an invasive pest with economic importance worldwide. Sex ratios during egg-hatching and a frequency of polyandry in single-female families were analysed to clarify the reproduction strategy of CPB, which was still known only in fragments. 1296 just hatching 1st instar CPB larvae were collected from 19 single-female families, of which 13 were random families collected from potato fields and 6 were families produced by laboratory farming of naturally fertilised females. All larvae were analysed to detect a sex using a qPCR-based method and to detect polymorphisms in genotypes of 9 microsatellite (SSR) markers. The bias in sex ratio in favour of females was confirmed using linear mixed-effects model in both experimental groups of families: field collections (*F* = 36.39*; P* = 0.0001) and laboratory farming (*F* = 13.74; *P* = 0.0139). The analysis of diversity in microsatellites proved the polyandry in all progenies as 73% of analysed segregation patterns did not match with the patterns expected for full-sib progenies; on average per locus, 46% of allelic and 49.7% of genotype ratios showed irregular segregation. Both findings contribute toward understanding CPB success rate as an invasive species, as the preferential bearing of females with polyandry has a great potential to keep fitness of progenies, to maintain and operate population diversity, and to accelerate the reproduction of the pest.

## Introduction

Strategies in sexual reproduction in beetles (Coleoptera) are various. Most species reproduce sexually and for some species asexual reproduction takes precedence^1^. The sexual reproduction in Coleoptera is controlled by several sex determination systems^2^, where the main indicators are the male heterogamety XY and X0^3^. The sexual reproduction of beetles generally maintains a genetic variation in progenies and, following Fisher’s principle, it is presumed to produce an equilibrated sex ratio 1:1 in population. Female-biased sex ratios, which are typically caused by genetic, cytoplasmic, or environmental factors, are known in many species or in their separate populations^4^, including Coleoptera. The genetic mechanisms, such as an arrhenotokous parthenogenesis typical for Hymenoptera, are very rare in Coleoptera (ambrosia beetle *Xylosandrus compactus* Eichhoff is exemplary)^5^. In contrast, the cytoplasmic factors, which are represented by endosymbiotic bacteria (in example of *Wolbachia pipientis* Hertig 1936^6^) are more frequent in beetles. In some species, the endosymbiont induces parthenogenesis (thelytoky) and cytoplasmic incompatibility^7^ as well as embryonic male killing^8^. On the other hand, wolbachia-mediated feminization of males, which is known in Lepidoptera^9^, is unknown in Coleoptera. The possible environmental factors affecting the slight increase in frequency of females can be represented by temperature and precipitation. A similar identification was made by Böcher and Nachman^10^ for a heteropteran species *Nysius groendlandicus* Zetterstedt. However, the specific mechanisms of how the sex ratios are modulated by environmental factors are unknown. Biased sexual ratios in population of adults represent an operational sex ratio (OSR), which is an effective reproductive ratio of both females and males^11^. The OSR influences specific sexual behavior in a population as well as the rate of sexual selection of males^12^, since the rarer males have a better opportunity to copulate. This can result in polyandry (mating of a female with multiple mates) which is well known in the red flower beetle *Tribolium castaneum* Herbst^13^ and prolongs lifetime offspring production by a female^14^. In such situations, it is observed that the proportion of various mates in progenies is not equivalent. These conflicts can be caused by cryptic female choice of sperm^15,16^ or by sperm competition resulting preferentially in last-male precedence^17^.

In the Colorado potato beetle (CPB, *Leptinotarsa decemlineata* Say, 1824), an invasive herbivorous pest of economic importance worldwide^18^, the sexual strategies are known only in fragments. The species is known to have the X0 system of sex determination^19^. The last-male precedence was confirmed by using various protein markers for this species^20^. A potential for wolbachia-mediated parthenogenesis, known in family *Chrisomelidae* and recently studied in CPB^21^, was never reported^22^. An existence of biased sex ratios and polyandry have not been studied in CPB populations, however, the tools to study them are available. To study the bias of the sex, the sexing method based on the copy number variation of X-linked loci in CPB, which we published recently^23^, can be used effectively. The polyandry can be studied using microsatellite markers developed by Grapputo^24^.

This work is aimed to study uncovered aspects of CPB reproduction strategy, which can be deemed important for the understanding of success of the species as an invasive pest of solanaceous plants. The research objectives were to characterise primary sex ratios and to evaluate an existence and rate of polyandry in CPB populations.

## Material and methods

### Collections of samples and preparation of progenies

Experimental 1st instar CPB larvae of the 1st generation originated from temporary laboratory farming and collections in potato fields. For laboratory farming, random naturally fertilised females were collected from experimental potato fields of the Crop research institute in localities Prague Ruzyne and Travcice, immediately after spring adults’ emergence. The females were individually placed in petri dishes with potato leaves and left to lay eggs. The egg colonies in petri dishes were left on the laboratory bench top to incubate under common laboratory temperature, light regime and humidity, until hatching of larvae. In experimental fields of the Potato research institute Ltd. and participating potato growers in Celakovice, Chlumin and Zabcice, single-female colonies of eggs were collected, transported to the laboratory, and incubated in the same way to obtain larvae. Females and eggs were collected from fields untreated by insecticides to maintain reproductive potential of females as well as integrity and completeness of progenies. Each experimental larva was separately placed in an 0.5 ml polypropylene tube, terminated in liquid nitrogen and kept in a freezer until DNA extraction. Information of localities with numbers of progenies and larvae are presented in Tables 1 and 4.

**Table 1:** Localities of collections of CPB larvae.

Locality	Region	GPS	Sampling	N_families_	N_larvae_
Celakovice	Central Bohemia Region	50.1603833N, 14.7500453E	Field collection	3	57
Chlumin	Central Bohemia Region	50.2891147N, 14.4492758E	Field collection	4	48
Prague Ruzyne	Prague the Capital City	50.0922525N, 14.3004383E	Lab. farming	1	77
Travcice	Central Bohemia Region	50.5032850N, 14.1897669E	Lab. farming	5	972
Zabcice	South Moravian Region	49.0115967N, 16.6025722E	Field collection	6	142

### DNA extractions

DNA of individual larvae was extracted using the CTAB method by Chen et al.^25^ modified by Sedláková et al.^23^. DNA samples were quantified with a NanoPhotometer (Implen, Germany) and diluted with PCR grade water (Sigma, Germany) to a concentration of 5 ng µL^−1^.

### Detection and statistical evaluation of diversity in microsatellites and polyandry detection

Ten SSR markers by Grapputo^24^ were used to evaluate the diversity of microsatellite loci in individual CPB families, evaluating families from laboratory farming. The SSR patterns of each mother were also evaluated to validate the alleles and genotypes detected, in order to exclude markers potentially producing false alleles. The markers were reorganized into two multiplexes. For mass evaluation using capillary electrophoresis, the forward primers were 5′ fluorescently labeled as recommended by author, however the revers primers were modified at 5′end by 5′GTTTCTT3′ tail by Brownstein et al.^26^ to support 3′ adenylation of amplicons. All modifications of primers, their placement to multiplexes, and final concentrations in PCR mix, are presented in Table 2. The mixes of both PCR multiplexes of volume 10 µl contained 5 ng of DNA, optimized concentration of all primers and 5 µl of 2× Multiplex PCR Master Kit (Qiagen, Germany). PCR was run in thermocycler C1000 (BioRad, USA) as follows: initial denaturation (95 °C, 10 min) followed by 35 cycles of denaturation (94 °C 30 s) with annealing (56 °C, 90 s) and extension (72 °C, 60 s), closed by final incubation step (60 °C, 80 min). The amplicons of multiplex 1 were diluted by PCR grade water in ratio 1:19 and amplicons of multiplex 2 in ratio 1:9. 1 µl of the diluted product was mixed with 12 µl of HiDi formamide (Applied Biosystems, USA) and 0.2 µl of size standard GeneScan LIZ600 (Applied Biosystems, USA), denatured in thermocycler (95 °C, 5 min) and analyzed by capillary electrophoresis Genetic Analyzer ABI PRISM 310 (Applied Biosystems, USA). Length polymorphisms of microsatellite markers were detected using the GeneMapper v 4.1 software (Applied Biosystems, USA). Allelic data were processed using GenAlEx 6.5 software^27,28^ to obtain general population data (Table 2) and information of the number and frequency of alleles observed in each progeny. Polymorphic information content (PIC) was calculated using Gene-Calc^29^. Number and frequency of genotypes were detected using simple data filters in MS Excel 2019 (Microsoft, USA). Both allelic and genotype frequencies for each locus were compared with expected full-sib ratios presented in Table 3 using χ^2^-test in MS Excel. Intensity of association (Φ) between genotype and allelic segregation was evaluated using the “2 × 2” association analysis in Dell Statistica software (Dell, USA). Statistical decisions were done at a standard significance threshold (α = 0.05).

**Table 2:** Design of multiplex PCR optimised by Grapputo^24^.

Primer	Sequences (5′–3′) and fluorescent labelling	Conc. [µM]	N_a_	Size range of alleles	H_o_	H_e_	PIC
**Multiplex 1**							
LdE11cF	**6-FAM**–GCGGCCAGATGTTATCAGTT	0.04	3	152–164	0.031	0.043	0.042
LdE11cR	GTTTCTTCACCGCGACTTCAAAGGTAT	0.04					
LdAC5-2F	**6-FAM**–CACTCTGGGGTCAAATAGAG	0.08	8	222–239	NA	NA	NA
LdAC5-2R	GTTTCTTTTCGAGTGACTTGTGTGTGT	0.08					
LdAC5-22F	**VIC**–CGTTTATGATTAGCATTTCTGA	0.04	3	152–164	0.786	0.642	0.565
LdAC5-22R	GTTTCTTACTTTCAATAAAAAGGTCGAT	0.04					
LdGA4-5F	**NED**–CCAGTTGATATTGAGAGAGA	0.08	5	206–226	0.424	0.441	0.405
LdGA4-5R	GTTTCTTCAAACGCACTCAGTACAAAA	0.08					
**Multiplex 2**							
LdA11bF	**VIC**–CAACGTACAGTGTGCTTCATTTG	0.06	4	169–175	0.248	0.465	0.407
LdA11bR	GTTTCTTTCAAGATTTGTTGCAGACATCA	0.06					
LdB8bF	**VIC**–TGCTCATTTCAAATATGGTTTTG	0.04	4	100–107	0.689	0.645	0.599
LdB8bR	GTTTCTTCCAACAGGTATCCAACAAACG	0.04					
LdE10eF	**PET**–ACAGCGTCCCTGTCACTTCT	0.08	3	128–134	0.339	0.354	0.292
LdE10eR	GTTTCTTCCCAGCGAGGTTTATTAGGA	0.08					
LdGA5-11F	**NED**–TTTGGTGGGTGTTTCTATTC	0.08	5	179–191	0.742	0.684	0.622
LdGA5-11R	GTTTCTTAAATGCGCCTGATGATAG	0.08					
LdGA4-18F	**PET**–GCTCGTCAAATCTAGGAAGA	0.20	5	210–217	0.371	0.601	0.519
LdGA4-18R	GTTTCTTAAGAATGAAATCCAGGAGAA	0.20					
LdGA5-30F	**6-FAM**–GTTTTCCATCATGATCCATT	0.08	5	181–186	0.358	0.731	0.683
LdGA5-30R	GTTTCTTATAGGAAGCAACGACCATC	0.08					

Results of general population analysis of the CPB collection are represented by number of detected alleles per locus (N_a_), size range of detected alleles, heterozygosity observed (H_o_) and expected (H_e_) and polymorphic information content (PIC).

**Table 3:** An overview of the expected segregation ratios of microsatellite loci in full-sibs of CPB.

Alleles per locus (obs.)	Mating (exp.)	Genotypes (exp.)	Frequency of alleles (exp.)
2	A_1_A_1_ × A_2_A_2_	A_1_A_2_	A_1_ = 0.50; A_2_ = 0.50
	A_1_A_1_ × A_1_A_2_	A_1_A_1_: A_1_A_2_	A_1_ = 0.75; A_2_ = 0.25
	A_1_A_2_ × A_1_A_2_	A_1_A_1_: 2A_1_A_2_: A_2_A_2_	A_1_ = 0.50; A_2_ = 0.50
3	A_1_A_2_ × A_3_A_3_	A_1_A_3_: A_2_A_3_	A_1_ = 0.25; A_2_ = 0.25; A_3_ = 0.50
	A_1_A_3_ × A_2_A_3_	A_1_A_2_: A_1_A_3_: A_2_A_3_: A_3_A_3_	A_1_ = 0.25; A_2_ = 0.25; A_3_ = 0.50
4	A_1_A_2_ × A_3_A_4_	A_1_A_3_: A_2_A_3_: A_1_A_4_: A_2_A_4_	Each allele = 0.25

Producing half-sibs, the polyandry should make significant deviations in frequencies of observed genotypes and alleles against those expected.

The polyandry was detected on a principle of the comparison of observed allelic frequencies and genotype segregation rates with the expected values defined by general principles of Mendelian inheritance and population genetics. The respected genetic limits for single-female full-sib progeny of diploids are as follows: (1) up to 4 various alleles and genotypes and up to 2 diverse homozygous genotypes can be detected in each locus, and frequencies of alleles and genotype segregation in each locus should associate with expected frequencies and ratios, respectively, which are presented in Table 3.

### Detection of sex of CPB larvae and statistical evaluation of sex ratios

The sex of larvae was detected using the method of CPB sexing by Sedláková et al.^23^. The general hypothesis of a balanced female-male ratio was tested at a standard significance threshold (α = 0.05) using linear mixed-effects model (LMM). Analyses were performed by *lmer* function^30^ from *lme4* package^31^ using R software version 4.2.0^32^. Two various models were designed for two independent experimental groups. The group of progenies from the field collections of eggs was analyzed using a model accounting for the random effects of mothers and locations (Eq. 1). The group of the laboratory reared families was analyzed by a model accounting for the random effect of mother only (Eq. 2).

1 $$nr{\text{-}}{individuals}_{ijk}=\alpha +{\beta }_{1}\times {sex}_{i}+{a}_{j}\left({b}_{k}\right)+{\epsilon }_{ijk} \; {\text{where}}\;{a}_{j}\;{\text{and}}\;{b}_{k} \sim N\left(0,{\sigma }^{2}\right) \; {\text{and}}\; {\epsilon }_{ijk}\text{ }N\left(0,{\sigma }^{2}\right)$$

*nr-individuals*_*ijk*_ is the number of individuals per *i*-th sex, *j*-th mother (n_*j*_ = 13) and *k*-th locality (*n*_*k*_ = 3), *sex*_*i*_ is a fixed effect (*i* = male, female), *a*_*j*_ is a random effect of *j-*th mother nested within *k-*th locality, *b*_*k*_ is a random effect of *k*-th locality and $\epsilon$_*ijk*_ is a residual error.

2 $${nr{\text{-}}individuals}_{ij}=\alpha +{\beta }_{1}\times {sex}_{i}+{a}_{j}+{\epsilon }_{ij} \; \mathrm{where} \; {a}_{j} \sim N\left(0,{\sigma }^{2}\right) \, \; {\text{and}} \; { \epsilon }_{ij}\sim N\left(0,{\sigma }^{2}\right)$$

*nr-individuals*_*ij*_ is the number of individuals per *i*-th sex and *j*-th mother (*n*_*j*_ = 6), *sex*_*i*_ is a fixed effect (*i* = male, female), *a*_*j*_ is a random effect of *j-*th mother and $\epsilon$_*ijk*_ is a residual error.

### Ethic approval

All of the experimental procedures were conducted in accordance with Czech legislation (Section 29 of Act No. 246/1992 Coll. on the protection of animals against cruelty, as amended by Act No. 77/2004 Coll.). We hereby declare that animal handling conducted in our study complies with the relevant European and international guidelines on animal welfare, namely Directive 2010/63/EU on the protection of animals used for scientific purposes and the guidelines and recommendations of the Federation of Laboratory Animal Science Associations. All experimental protocols were approved by the Czech University of Life Sciences Prague, Faculty of Agrobiology, Food and Natural Resources of the Czech Republic and Institutional and National Committees. Collections of larvae and adults in potato fields were done by the approval of owners of the fields.

## Results

### Larvae sampling and DNA extractions

**Figure 1:**
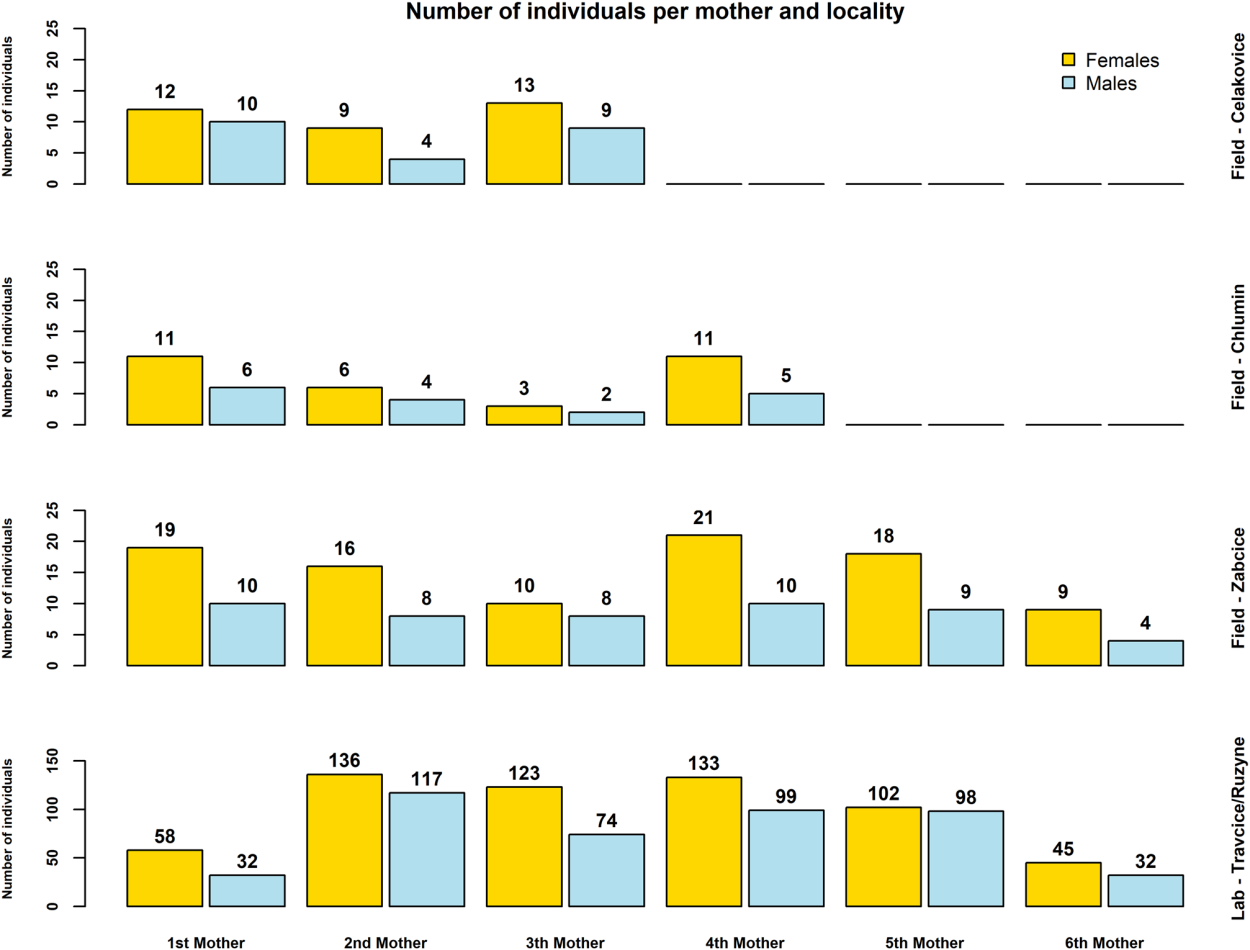
Results of sexing of larvae. The numbers of females and males per progeny are presented for each locality separately. The 6th mother in the cluster Lab-Travcice/Ruzyne represents results of sexing of the progeny Prague Ruzyne 1. Designed in R software version 4.2.0.

Total number of 1296 1st instar CPB larvae were obtained and analysed genetically. They belonged to 19 single-female progenies, of which six progenies were produced by laboratory farming and 13 progenies were collected from potato fields. The eggs hatched in laboratory almost completely, up to three unhatched eggs were observed in each colony. Detailed numbers of hatched larvae per sample can be seen in Fig. 1. The present method of DNA extractions of the larvae offered DNA of sufficient quantity (25.8 ng µl^−1^, SD = 10.2 ng µl^−1^) and quality (ratio of A260/A280 was 1.89 in average) for all analyses.

### Sex detection of larvae and sex ratio in progenies

Across the 19 evaluated sex ratios, 755 females and 541 males were detected. Results of sex detection and segregation are presented in Fig. 1. The data shows that both sexes occurred regularly in all progenies, however the numbers of females and males in each family indicated a female-biased sex ratio. LMM analysis of datasets confirmed a general bias in sex ratio in both field-collected clutches (*F* = 36.39; *P* = 5.9 × 10^–5^) and laboratory-reared clutches (*F* = 13.74; *P* = 0.0139). The random effect of locality on sex ratio could not be accounted in the second group for limited data from locality Prague Ruzyne. The validity of the results is documented by the graphical output of the assumptions diagnostics for both datasets (Supplementary Fig. S1 and S2).

### Microsatellite analysis

The redesigned PCR multiplexes produced quality genetic profiles in all evaluated markers. To confirm the validity of all alleles, genotypes of laboratory reared progenies were compared with genotypes of their mothers. At least one maternal allele per each locus was detected in linked offspring genotype, except for the marker LdAC5-2. This marker produced false positive alleles and has therefore been excluded from statistical evaluations. Nine loci in 19 progenies offered 171 genotype segregation ratios and 171 allelic ratios. The ratios were compared with segregation ratios expected for full-sib progenies presented in Table 3 using χ^2^ test. The number of alleles and genotypes are presented with p-values of χ^2^ test in Tables 4 and 5 respectively. The maximal expected number (MEN) of alleles was never exceeded, however 49.7% of evaluated allelic ratios differed significantly from the expectations (*P* < 0.05). In contrast, 70% of genotype segregation ratios were abnormal, of which 21% exceeded MEN of genotypes per locus and 49.7% shown abnormal segregation only. Such abnormalities in both allelic and genotype ratios indicate that the half-sib structure of progeny is the result of polyandry.

**Table 4:** Numbers of alleles per SSR locus detected in single-female families and χ^2^ probability of agreement with the expected ratio: 4 alleles in ratio 1:1:1:1, 3 alleles in ratio 2:1:1, 2 alleles in ratio 1:1 or 3:1.

Progeny	LdAC5-22	LdE11c	LdGA4-5	LdGA5-30	LdB8b	LdA11b	LdGA5-11	LdE10e	LdGA4-18
Celakovice 1	3 **0.005**	1 NA	3 **0.000**	3 0.654	3 **0.000**	3 0.654	3 **0.000**	2_(3:1)_ 0.451	3 **0.000**
Celakovice 2	3 0.264	1 NA	1 NA	2_(3:1)_ **0.026**	4 **0.001**	2_(3:1)_ **0.000**	2_(3:1)_ **0.000**	2_(1:1)_ 0.442	3 0.070
Celakovice 3	3 **0.000**	2_(1:1)_ 0.054	4 **0.000**	2_(3:1)_ **0.026**	3 **0.000**	3 **0.000**	3 **0.000**	1 NA	3 0.895
Chlumin 1	3 **0.036**	1 NA	2_(3:1)_ 0.521	2_(3:1)_ 0.054	3 **0.000**	2_(1:1)_ **0.026**	3 **0.000**	2_(3:1)_ 0.199	3 **0.000**
Chlumin 2	2_(1:1)_ 0.135	1 NA	2_(3:1)_ 0.248	3 **0.009**	3 **0.000**	2_(1:1)_ 0.317	2_(3:1)_ 0.248	2_(3:1)_ 1.000	2_(1:1)_ 0.317
Chlumin 3	3 0.354	1 NA	2_(3:1)_ **0.000**	3 **0.000**	4 **0.000**	2_(3:1)_ 0.209	4 0.556	2_(3:1)_ 0.209	3 0.753
Chlumin 4	3 **0.030**	1 NA	4 **0.000**	4 **0.000**	4 **0.000**	2_(3:1)_ 0.470	3 0.372	3 **0.000**	3 **0.001**
Zabcice 1	3 **0.000**	1 NA	3 **0.000**	2_(1:1)_ 0.168	4 **0.001**	2_(3:1)_ 0.550	3 **0.000**	2_(1:1)_ 0.168	2_(1:1)_ 0.730
Zabcice 2	3 **0.010**	1 NA	4 **0.000**	4 **0.001**	4 **0.000**	2_(3:1)_ **0.004**	4 **0.000**	2_(3:1)_ **0.016**	3 0.353
Zabcice 3	3 0.125	1 NA	3 0.763	4 **0.000**	3 **0.000**	2_(3:1)_ **0.010**	3 0.071	2_(3:1)_ 0.199	3 0.231
Zabcice 4	2_(1:1)_ 0.747	1 NA	2_(1:1)_ 0.747	3 0.791	3 0.974	2_(3:1)_ **0.000**	2_(3:1)_ 0.166	1 NA	3 0.856
Zabcice 5	2_(3:1)_ **0.019**	2_(3:1)_ **0.000**	2_(3:1)_ 0.831	3 **0.001**	3 0.424	2_(3:1)_ 0.831	3 0.787	2_(3:1)_ 0.285	2_(3:1)_ 0.831
Zabcice 6	3 0.264	1 NA	2_(3:1)_ **0.026**	3 **0.000**	4 **0.000**	2_(1:1)_ 0.124	2_(1:1)_ 1.000	2_(1:1)_ 0.442	2_(3:1)_ **0.026**
Prague Ruzyne 1	3 0.666	1 NA	2_(3:1)_ 0.139	3 **0.001**	4 **0.000**	2_(3:1)_ **0.026**	3 0.085	2_(3:1)_ **0.001**	2_(3:1)_ 0.076
Travcice 1	3 **0.000**	2_(3:1)_ **0.000**	3 **0.000**	3 **0.018**	4 **0.000**	2_(3:1)_ **0.004**	4 **0.006**	2_(3:1)_ **0.019**	4 **0.000**
Travcice 2	2_(1:1)_ 0.278	1 NA	2_(3:1)_ 0.397	3 0.087	3 **0.000**	2_(3:1)_ **0.003**	2_(3:1)_ 0.644	2_(3:1)_ **0.000**	3 0.249
Travcice 3	3 **0.000**	3 **0.000**	3 **0.010**	3 0.263	4 **0.029**	2_(3:1)_ 0.269	3 **0.000**	2_(3:1)_ 0.219	3 **0.035**
Travcice 4	3 0.206	2_(3:1)_ **0.001**	3 **0.000**	4 **0.000**	3 **0.002**	2_(3:1)_ **0.009**	3 **0.000**	2_(3:1)_ 0.852	2_(1:1)_ 0.096
Travcice 5	3 **0.023**	3 **0.000**	2_(3:1)_ **0.000**	3 **0.000**	2_(3:1)_ 0.586	2_(3:1)_ 0.355	3 0.980	2_(3:1)_ 0.828	2_(1:1)_ 0.053

χ^2^ values are missing for when only one allele per locus was observed in homozygous genotype (NA). Bold style font indicates significant discrepancy of ratios, which confirms polyandry.

**Table 5:** Number of genotypes per SSR locus detected in single-female families and χ^2^ probability of agreement with the expected genotype ratio: 4 genotypes in ratio 1:1:1:1, 3 genotypes in ratio 2:1:1, 2 genotypes in ratio 1:1.

Progeny	LdAC5-22	LdE11c	LdGA4-5	LdGA5-30	LdB8b	LdA11b	LdGA5-11	LdE10e	LdGA4-18
Celakovice 1	3 0.078	1 NA	3 **0.000**	4 **0.003**	4 **0.000**	4 **0.005**	2 **0.000**	2 0.192	3 **0.000**
Celakovice 2	2 **0.021**	1 NA	1 NA	2 **0.000**	6 **NA**	2 **0.000**	2 **0.000**	3 **0.070**	4 **0.000**
Celakovice 3	3 **0.000**	3 0.744	6 **NA**	2 **0.000**	5 **NA**	4 **0.000**	3 **0.039**	1 NA	4 0.898
Chlumin 1	4 **0.000**	1 NA	2 0.267	2 **0.000**	2 **0.000**	3 **0.004**	3 **0.000**	2 **0.026**	3 **0.000**
Chlumin 2	2 **0.000**	1 NA	2 **0.046**	3 0.368	2 **0.000**	3 **0.000**	2 **0.046**	3 **0.000**	3 0.135
Chlumin 3	4 **0.000**	1 NA	2 **0.000**	3 **0.001**	5 **NA**	2 **0.030**	4 0.085	2 **0.030**	3 **0.000**
Chlumin 4	2 **0.000**	1 NA	5 **NA**	6 **NA**	4 **0.000**	3 **0.044**	5 **NA**	3 **0.009**	4 **0.000**
Zabcice 1	2 **0.000**	1 NA	5 **NA**	3 **0.048**	6 **NA**	2 **0.000**	5 **NA**	3 0.364	3 0.837
Zabcice 2	5 **NA**	1 NA	5 **NA**	6 **NA**	5 **NA**	2 **0.000**	6 **NA**	3 **0.009**	5 **NA**
Zabcice 3	3 0.062	1 NA	4 **0.000**	6 **NA**	3 **0.000**	3 **0.000**	4 **0.011**	3 0.157	4 **0.011**
Zabcice 4	2 **0.000**	1 NA	3 **0.015**	2 **0.333**	4 **0.005**	2 **0.000**	2 **0.024**	1 NA	4 **0.003**
Zabcice 5	2 **0.000**	2 **0.000**	2 0.711	4 0.295	2 0.064	2 0.711	4 0.187	2 0.064	2 0.711
Zabcice 6	5 **NA**	1 NA	3 **0.000**	3 **0.000**	4 **0.000**	3 **0.000**	3 0.744	3 0.070	3 **0.004**
Prague Ruzyne 1	4 0.119	1 NA	3 **0.000**	5 **NA**	7 **NA**	5 **NA**	3 0.081	2 **0.000**	2 **0.002**
Travcice 1	5 **NA**	2 **0.000**	3 **0.000**	5 **NA**	4 **0.008**	2 **0.000**	7 **NA**	2 **0.000**	6 **NA**
Travcice 2	3 **0.000**	1 NA	2 0.142	4 0.431	4 **0.000**	5 **NA**	2 0.505	2 **0.000**	5 **NA**
Travcice 3	4 **0.000**	3 **0.000**	5 **NA**	6 **NA**	6 **NA**	3 0.153	4 **0.000**	3 0.212	2 **0.000**
Travcice 4	5 **NA**	3 **0.000**	5 **NA**	6 **NA**	3 0.080	3 **0.005**	3 **0.000**	2 0.747	3 **0.014**
Travcice 5	3 0.112	3 **0.000**	3 **0.000**	5 **NA**	2 0.345	2 0.109	5 **NA**	2 0.706	3 0.591

χ^2^ values are missing when uniform homozygote or more than 4 genotypes per locus were detected (NA). Bold style font indicates significant discrepancy of ratios, which confirms polyandry.

**Figure 2:**
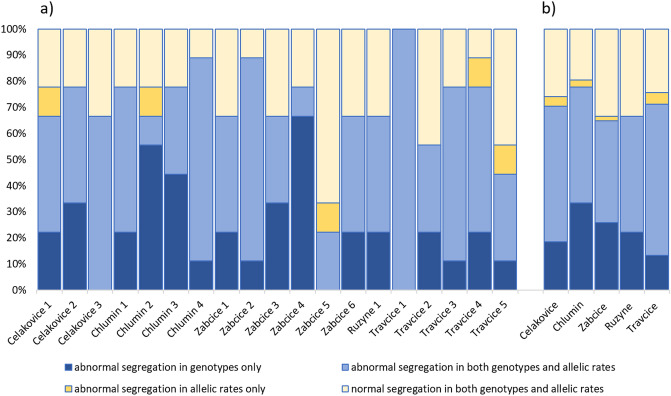
Genotype and allelic segregation rates of microsatellite loci. The results of genotype segregation are associated with the allelic segregation and presented for each CPB progeny (**a**) and each locality (**b**). The polyandry of various intensity was detected in all progenies. Designed in MS Excel.

Regardless of the offspring number in progeny, the abnormal segregation ratios were dispersed equally across all progenies and loci (Fig. 2). On average per progeny, 4.5 (50%) allelic and 6.3 (73%) genotype irregular segregations were detected. Positive association between irregular allelic and genotype segregations (Φ = 0.528) was observed. The Φ-values increased with number of individuals per progeny, where the highest association was observed in Travcice and Prague Ruzyne (Φ = 0.617), followed by Zabcice (Φ = 0.568), Celakovice (Φ = 0.562), and Chlumin (Φ = 0.371).

## Discussion

### Sex ratios in CPB population

Our experiment shows that the CPB populations produced egg clutches with a female-biased sex ratio. Any other works directly aimed at the study of natural sex ratios in CPB could not be found, however, the progenies unbalanced in sex are relatively common in Coleoptera and insects generally^4,6^. We consider our result important for better understanding the invasiveness of CPB, because the female-biased sex ratios can produce more females directly responsible for spreading of the pest. The lower frequency of males in population helps to reduce a selection pressure on males^12^ and can support a wider sexual promiscuity in CPB population^13^ (in our experiment represented by a polyandry). Vahl et al.^33^ experimentally established a better fertilisation probability of CPB females with an increasing density of males in environment, however the female-biased sex ratios near to 2:1 kept the fertilisation probability on satisfactory value *P* = 0.5. We assume, that both phenomena confirmed by us have the same importance as generally accepted ecological adaptations and stress tolerance of the species^18,34^.

It is important to discuss the timing and way of sampling regarding the present methods. The study of sex ratios in later developmental stages^10^ is suitable to evaluate operational sex ratios^11^. However, it is not informative enough in terms of sex bias caused by internal effects of female (discussed below). The OSR of adults is a result of primary sex ratio development during an ontogenesis, which can be affected generally by an insecticides-mediated sex-specific selection. Such selection, under effect of insecticide DDT, is well known in *Drosophila melanogaster* Meigen^35^. Sexually unspecific factors reducing populations of CPB are as follows: a use of insecticides, predators (in example Coccinellidae), pathogenic fungi and bacteria, parasitic nematodes and an overwintering. The overwintering in CPB is especially dependent on content of lipids in offspring of insecticide resistant parents^34^ and on temperature of soil^36^. The objective of the present research was to study sex ratios in time of oviposition, which reflects a primary set up of population influenced only by internal, genetic and cytoplasmic, factors formulated by Cook^4^. The used way of sampling and sexing enabled us to reliably analyze sex ratios of CPB and to remove most above-mentioned environmental factors leading to OSR, including the pesticides, which were not used in subjected localities immediately prior to sampling. Both methods of sampling used offered relatively comparable results as shown by the LMM analysis. However, the use of larger numbers of more numerous laboratory-bred families is recommended to improve the strength of statistical evaluation.

A general shift in the primary sex ratio in favour of females was found. This was probably caused by a specific effect of female combined with an effect of locality, as suggested by the LMM analysis of data within the group of progenies collected in fields. Nevertheless, only various hypothetical ways of sex ratio modification in these CPB population samples can be discussed. A frequency of males in the progenies practically ruled out a parthenogenesis (thelytoky). In Coleopters, it is usually Wolbachia-mediated^7,8^, but absent in CPB, as has been relatively recently reported for a geographically-near Polish CPB population^21^. The significant effect of female on the sex ratio detected by LMM, indicates hypothetical genetic control by a male-specific lethal factor which should block development of male zygotes or early embryos. Such male-killing factors are known in several Dipteran species^37^ (e.g. the *Nix* sex-determining factor), and they have a potential of use in a pest management^38^. These factors for the CPB have not been reported, however, considering the observed segregation ratios in the experimental progenies, two hypothetical models of the primary sex ratio modification arise. The first scenario presumes a single autosomal recessive male-killing factor, which selects out 1/8 of offspring (25% of recessively homozygous males) in progeny of two heterozygotes and results in the female-biased sex ratio 4:3. The second scenario presumes a recessive X-linked male-killing factor, which reduces the sex ratio in progeny of heterozygous female on 2:1, as half of males (recessive hemizygotes) is selected out. Being effective only in recessive early male-embryos, these factors should be maintained stably in populations through heterozygous females (X-linked factors) or through heterozygotes generally (autosomal factors). Nevertheless, further research to confirm this hypothesis is required. Raw segregation ratios in Table 4 suggest an existence of both models distributed in populations, however the data range, especially in progenies from field collections, is insufficient to complete a reliable verification of these hypotheses.

### Diversity in microsatellites and polyandry

The polyandry is frequent in populations of animals and helps to maintain the genotype variation in progenies^39^. Some conditions and effects of polyandry in CPB has been partly studied by Boiteau^40^, who identified partial male precedence and a need of CPB females to copulate with at least three independent males to fill their reproduction potential. More recently, the last-male precedence was experimentally confirmed using neutral enzyme markers in a controlled mating of female with two diverse males^20,41^. However, these approaches could not identify both a frequency of polyandry in the field CPB populations and an effectiveness of the last-male precedence, in more realistic multiple-mating systems. The problems of experiments based on the artificial mating of preselected individuals were discussed extensively by Arnaud^42^.

The main objective was to study a polyandry range of females fertilised naturally in field conditions. Our approach assumed that the polyandry produces the half-sib progenies, which is possible to study using neutral microsatellite markers. As the results showed, the use of slightly modified microsatellite panel by Grapputo^24^ allowed to compare the observed segregation ratios with the expected full-sib ratios following the general principles of Mendelian inheritance and population genetics. The modification of microsatellite panel was necessary, because some loci repeatedly failed in amplification. The 5′end modification of revers primers using 5′GTTTCTT3′ tail by Brownstein et al.^26^ significantly reduced stuttering of alleles and improved their automatic detection using GeneMapper 4.1. The knowledge of maternal alleles in laboratory reared progenies from Travcice and Ruzyne helped to identify considerable number of false alleles produced by marker LdA5-2, which led to excluding the marker from the analysis. However, this experience confirms that it is almost impossible to identify the false positive alleles in the population analysis of unrelated individuals without previous tests of newly generated microsatellite markers on the amplification instability.

The set of 9 remaining markers gave reasonable data to resolve the critical question of polyandry occurrence in CPB reproduction. The polyandry was confirmed in all evaluated progenies because 73% of segregation ratios did not associate with full-sib expectations. The phenomenon is presumed to be common in all CPB populations independently on environmental conditions. The following query was based on how many males participate on progeny of a female. The following problems that need to be solved have been identified: the number of detected alleles was too low (up to 4 alleles were detected per locus in each offspring) and no available software is able to solve such a difficult prognostic task without the genetic profiles of all potential parents. Another problem is also the low intra-locus variability allowing frequent mating of identical genotypes and the existence of discussed last-male precedence. It is assumed that the only solution to this problem is to use a haplotype analysis to identify male- and female-specific linkage groups of markers, where the specific haplotype clearly identifies the number of males on the background of two female haplotypes inherited by all offspring.

In conclusion, although evidence has been presented for the primary female-biased sex ratio and regular occurrence of polyandry in CPB reproduction strategy, important questions have yet to be answered. The mechanisms of sex ratio modification should be prospected and studied in detail. Furthermore, a system of haplotype analysis of CPB should be developed and applied to clarify specific details of polyandry.

## Supplementary Information

Supplementary Figure S1.

Supplementary Figure S2.

## Data Availability

Unpresented datasets generated and analyzed during the current study are available from the corresponding author on reasonable request.
