# Supplementary material for: Bias in sex ratios and polyandry rate in reproduction of Leptinotarsa decemlineata
Source: Sci Rep. 2022 Dec 14;12:21637. doi: 10.1038/s41598-022-26177-z (PMC9751100; doi:10.1038/s41598-022-26177-z)
Supplement: Supplementary file 2 — Supplementary Figure S2. [file 41598_2022_26177_MOESM2_ESM.pdf]

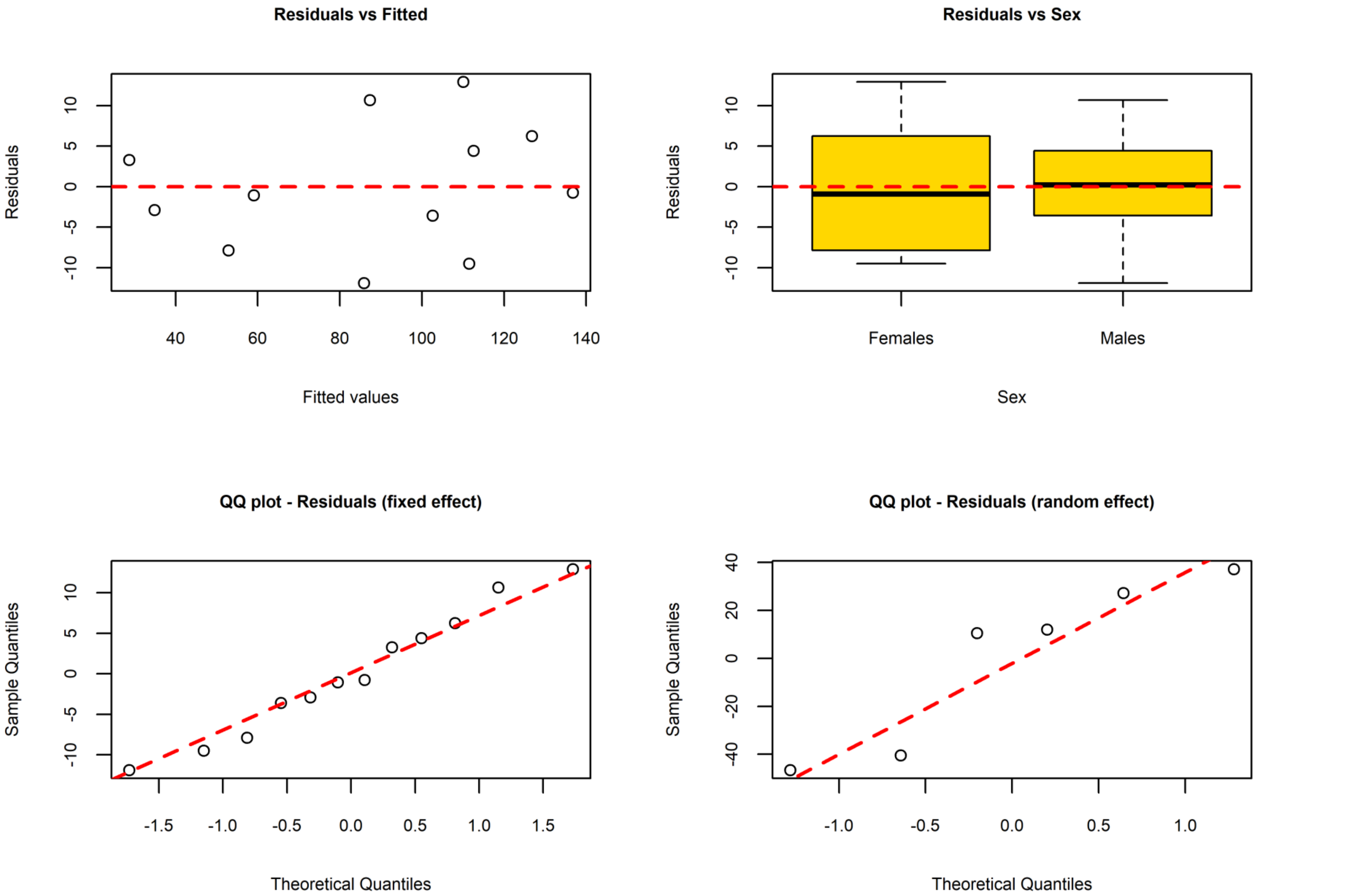

Figure S2. Validation of model assumptions by analysis of residuals in the dataset of laboratory farming. Homogeneity and independence were checked by plotting residuals against fitted values (upper left graph) and sex grouping variable (upper right graph). Normality of residuals was verified for both fixed and random effects by quantile-quantile graphs (bottom graphs).
